# The PaO_2_/FiO_2_ is a weak predictor of mortality for critically ill patients: limitations of an easy-to-calculate score

**DOI:** 10.62675/2965-2774.20260399

**Published:** 2026-03-16

**Authors:** Susana Mendes Fernandes, Rob Mac Sweeney, João Gonçalves Pereira

**Affiliations:** 1 Universidade de Lisboa Faculdade de Medicina Clínica Universitária de Medicina Intensiva Lisboa Portugal Clínica Universitária de Medicina Intensiva, Faculdade de Medicina, Universidade de Lisboa, Lisboa, Portugal.; 2 Royal Victoria Hospital Regional Intensive Care Unit Belfast Northern Ireland Regional Intensive Care Unit, Royal Victoria Hospital - Belfast, Northern Ireland.; 3 Hospital Vila Franca Xira Intensive Care Department Vila Franca Xira Portugal Intensive Care Department, Hospital Vila Franca Xira - Vila Franca Xira, Portugal.

Acute respiratory failure is a common cause for intensive care unit (ICU) admission. Around 40% of all patients admitted to the ICU will receive invasive mechanical ventilation, and around 10% of all ICU admissions will develop acute respiratory distress syndrome (ARDS).^(1)^ Respiratory failure is independently associated with a worse prognosis in mixed cohorts of patients admitted to the ICU. The arterial partial pressure of oxygen (PaO_2_) and fraction of inspired oxygen (FiO_2_) ratio (PFR) is easily calculated and serves as a common standard for assessing the severity of respiratory failure. Overall, it is also known that the lower the PFR, the higher the mortality, as evaluated in multiple observational trials.^(2)^ Given this association with mortality as a continuous variable, PFR is frequently used to assess patient prognosis and to categorize risk. This has led to PFR being incorporated into the ARDS severity criteria.^(3)^ In addition, PFR is used to guide treatment decisions according to the inclusion criteria used in trials: use of muscular relaxants (PFR below 100 - 150),^(4)^ proning patients (PFR below 150),^(5)^ recommending venovenous extracorporeal membrane oxygenation (VV-ECMO) support (PFR below 80). Despite this, observational data suggest that PFR cut-offs are unclear, and there is no clear value to guide prognosis or predict mortality.

In this issue of *Critical Care Science*, Ramanan et al.^(6)^ aimed to identify the optimal PFR for prognosticating mortality in the ICU, in the hospital, and at 6 months, using an extensive binational quality database from Australia and New Zealand. They included more than 600,000 patients admitted to the ICU, 51% with respiratory dysfunction, and identified a PFR of 230 as an optimal cut-off to discriminate mortality. Nevertheless, the area under the curve (AUC) was below 0.7, indicating a poor ability of PFR to predict mortality. Notably, PFR was not significantly better in pre-specified subgroups, namely patients explicitly admitted for acute respiratory failure, those supported with invasive ventilation, or those receiving VV-ECMO. Interestingly, the ability of day one PFR to predict mortality for patients supported with VV-ECMO was no better than flipping a coin.

For patients with acute respiratory failure, the authors found a lower PFR cut-off of approximately 180. This difference is interesting, as it suggests that context can significantly alter the PFR behavior. However, it is an even lower PFR, below 150, that triggers the introduction of more aggressive treatment, such as proning, neuromuscular blocking agents, or VV-ECMO, in invasive mechanically ventilated patients, consistent with the idea that a PFR like 230 would have little predictive value.

In fact, PFR has long been challenged as a reliable marker for acute respiratory severity.^(7)^ A lower ratio has been associated with a worse prognosis, and it can help characterize respiratory failure. Nevertheless, this correlation is not strong. Other markers of respiratory failure can also be considered, such as the peripheral capillary oxygen saturation (SpO_2_)/FiO_2_, especially in less resourced settings; the oxygenation index; and time-weighted PaO_2_. Besides, PFR kinetics in the first 24 hours have been used to identify and exclude patients with temporary respiratory failure (i.e. atelectasis) in numerous trials (ACURSY, PROSEVA, ART, EOLIA). In addition, the context in which respiratory failure occurs is a major determinant of prognosis (age, prior comorbidities, type of acute illness), along with the patient's tolerance to hypoxemia and hypercapnia. The PFR is only a small piece in prognostic determination. An elevated dead space ventilation, corrected minute ventilation, or ventilatory ratio, has also been shown to have a stronger association with mortality.^(8)^

**Figure 1:**
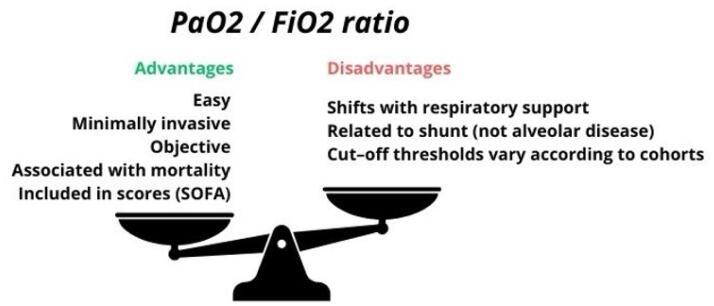
Pros and cons of the use of the PaO_2_/FiO_2_ to identify and classify respiratory failure.

The PFR depends on FiO_2_, the level of positive end-expiratory pressure (PEEP), and the arterial-venous oxygen extraction ratio and does not consider the mechanism of hypoxemia (Figure 1).^(9)^ A poor response to oxygen, due to pulmonary shunt, may result in a very low PFR, even in a pathology with a relatively good prognosis and easy resolution, such as atelectasis. In cardiogenic acute pulmonary oedema, a PFR of 100 might easily increase to 300 and lead to quick mechanical ventilation weaning. On the other hand, some interstitial diseases might lead to a relatively mild decrease in PFR, but with a high cost in lung compliance and ventilatory capacity, and a worse prognosis. The PFR cannot have the same meaning in all these settings.

The best PEEP and response to PEEP may lead to significant differences in PFR, usually decreasing shunt and increasing dead space. A positive response to PEEP has long been known to be associated with a better prognosis.^(10)^ Finally, the worst PFR in the first 24 hours may be reflective of pre-resuscitation pathophysiology and not necessarily reflect response to treatment or the way the support is being managed.

Altogether, this leads in daily practice to rapid shifts in PFR within a couple of hours after lung recruitment, either with changes in lung support or in a patient's position, and these are not linked to prognosis.

The PFR is easy to use and calculate, and it serves as a basis for assessing the severity of patients with acute respiratory failure. However, it has only moderate predictive ability for mortality. Besides, it cannot be interpreted independently of the respiratory support and patient context, as recently discussed in a rational supporting the revision of the Sequential Organ Failure Assessment (SOFA) score.^(11)^ Currently, it is maintained as the only marker of oxygenation severity, with slightly different cut-offs and with the valuation of the respiratory support received and of the clinical circumstances. For instance, be careful with evaluations immediately after suctioning.^(12)^ However, when assessing patients with respiratory failure, other markers of respiratory failure can be used in combination to characterize prognosis and to better identify severe patients who require more invasive treatment, or even to enrich trials with a less heterogeneous population. However, guiding interventions based on different markers of respiratory failure has not yet been proven to improve prognosis or the individualization of therapies. Careful implementation of these markers is needed.^(13)^

The PFR within 24 hours has a limited ability to predict progression or mortality from respiratory failure.^(14,15)^ Pragmatically, however, the worst PFR value in the first 24 hours is frequently collected in observational data. By choosing this time point, Ramanan et al.^(6)^ study highlights the limits of the predictive value of this measure.
